# Effects of Metabolic Syndrome and Its Components on the Prognosis of Endometrial Cancer

**DOI:** 10.3389/fendo.2021.780769

**Published:** 2021-12-16

**Authors:** Xiao Yang, Xingchen Li, Yangyang Dong, Yuan Fan, Yuan Cheng, Lirong Zhai, Shuyi Zhang, Jingyi Zhou, Jianliu Wang

**Affiliations:** Department of Obstetrics and Gynecology, Peking University People’s Hospital, Beijing, China

**Keywords:** endometrial cancer, metabolic syndrome, clinicopathological characteristics, overall survival, recurrence-free survival

## Abstract

**Objective:**

To explore the effects of metabolic syndrome (MetS) on the prognosis of endometrial cancer (EC) and to identify key components of MetS associated with EC.

**Methods:**

A total of 506 patients surgically diagnosed with EC were analyzed in this study. These patients were diagnosed with EC in the Department of Obstetrics and Gynecology at the People’s Hospital of Peking University between 2010 and 2016. The follow-up time was cut off at December 2019. MetS was characterized based on standards provided by the Chinese Diabetes Society in 2004.

**Results:**

Among the 506 EC patients analyzed, 153 patients were diagnosed with MetS. MetS patients were more likely to be older and postmenopausal. MetS was positively related to tumor grade, stage, LNM, LVSI, and MI. The univariate analysis showed that MetS was closely related to the OS (HR = 2.14; P = 0.032) and RFS (HR = 1.80; P = 0.045) of EC patients. K–M analysis also indicated that EC patients with MetS had shorter OS and RFS than EC patients without MetS. More specifically, patients that had ≥3 components showed a worse outcome compared with patients only having 0 or 1–2 components (P <0.05). In the multivariate-adjust model, after adjusting for age, histotype, tumor grade, and stage, HDL-C was found to be associated with increased risk of death related to EC (HR = 2.2, P = 0.034). However, MetS did not significantly correlate with this. ROC analysis revealed that the area under the ROC curve of combined factors (HDL-C + grade + stage) was better than traditional stage or grade at 1-, 3-, and 5-year survival rates. From this, a nomogram based on HDL-C, grade, and stage was constructed to predict survival of EC patients. Calibration curve analysis and decision curve analysis (DCA) showed the nomogram we constructed could better predict the survival of EC patients.

**Conclusion:**

MetS is closely related to poor prognosis in EC patients. The prevalence of individual MetS components increase with worse outcomes in EC patients. A nomogram based on HDL-C, grade, and stage has good ability to predict survival of EC patients.

## Introduction

Endometrial cancer (EC) is one of the most common gynecological malignancies. The latest cancer statistics from the SEER data showed that the estimated new EC cases in the United States increased by 66,570, and the estimated deaths increased by 12,940 in 2021, and the incidence rate was fourth among female malignant tumors and sixth in terms of deaths (1, 2). With lifestyle changes and the increased incidence of metabolic diseases (obesity, diabetes, and hypertension), the incidence and mortality rates of EC has been increasing worldwide. This EC incidence rate is expected to increase to 42.13 cases per 100,000 in the United States by 2030 (3). The mortality rates of EC increased 21% from 1999 to 2016 in the United States (4). Early stage EC patients show a more favorable prognosis, while advanced stage patients or cases of recurrence show a five-year survival rate less than 50% (5, 6). Therefore, it is of great clinical significance to explore the factors affecting the prognosis or recurrence of EC.

Recently, many risk factors have been linked to the occurrence of EC, such as obesity, diabetes and hyperinsulinemia. Epidemiological studies showed that the risk of EC was 2.45-fold higher in overweight patients (BMI ≥25 kg/m^2^) and 2.12-fold higher in diabetic patients (7). In addition, a sedentary lifestyle, Lynch syndrome, nulliparity, early menarche, and anovulatory conditions were also found to be potential risk factors for EC. Obesity-related insulin resistance is also a key factor associated with EC (8). At the same time, insulin resistance also leads to diabetes. Thus, obesity and diabetes may have common factors related to EC. It is well known that insulin directly promotes cell proliferation through the PI3K/Akt and Ras/MAPK pathways (9). There are many studies continually confirming that metabolic syndrome (MetS) consisted of obesity, diabetes/hyperglycemia, insulin resistance, hyperlipidemia, hypertension, and other metabolic abnormalities is closely related to the increased risk of various cancers, including prostate, colorectal and breast cancers (10–12). Also, MetS has been considered an important risk factor for EC. A recent meta-analysis showed that MetS diagnosed according to the NCEP-ATP III and IDF standards was closely related to an increased risk for EC (ORs) = 1.62 and ORs = 1.45, respectively (13). In addition, a Canadian population-based study showed that MetS was closely related to poor survival and disease-free survival in EC patients (14). However, few studies have explored the effects of MetS and its components on the prognosis of EC based on the Chinese population.

In our study, to evaluate the association between MetS and EC, we firstly explored the association between MetS and clinicopathological characteristics of EC patients. Then, we studied the effects of both MetS as a whole and its individual components on the prognosis of EC to provide new evidence for the association between MetS and EC. We also aimed to identify the key components of MetS associated with EC.

## Methods

### Patients Clinical Data

A retrospective study was performed that included 560 patients surgically confirmed to have EC at the Department of Obstetrics and Gynecology, People’s Hospital of Peking University between 2010 and 2016. Fifty-four patients were excluded from this study due to a family history of malignancy or missing data. MetS clinical data, clinicopathologic characteristics, and general patient information were collected. The recurrence and survival status of each patient was recorded, and the overall survival (OS) and recurrence-free survival (RFS) times were calculated. December 2019 was used as a cut-off for follow-up time. The study protocol was approved by the ethics committee of the Peking University People’s Hospital (2015PHB116-01).

### Defining MetS

MetS was defined based on the 2004 Chinese Diabetes Society standard, which explains that three or more of the following conditions must be present in a patient: 1) Overweight and/or obese: BMI ≥25.0 kg/m^2^; 2) Hyperglycemia: fasting blood glucose ≥6.1 mmol/L and (or) 2 h PG ≥7.8 mmol/L, or (and) those diagnosed as diabetic and were being treated; 3) Hypertension: blood pressure ≥140/90 mmHg, or (and) those diagnosed as having hypertension and were being treated; and 4) Dyslipidemia: Fasting triglycerides ≥1.69 mmol/l and/or HDL-C <0.9 mmol/l for males and <1.0 mmol/l for females.

### Statistical Analysis

The clinical statistical analysis was performed using EmpowerStats (http://www.empowerstats.com/). A *P <*0.05 was considered as statistically significant. The odds ratio (OR), hazard ratios (HRs), and 95% confidence intervals (CIs) were also calculated. Kaplan–Meier (K–M) survival curves were generated using Graphpad Prism 8.0. The time-dependent receiver operating characteristic (ROC) curve was analyzed using the “survivalROC” package in R. The nomogram was constructed using the “regplot” package in R. Calibration curves and decision curve analysis (DCA) were performed to evaluate the prediction accuracy of the prognostic model.

## Results

### Patient Clinicopathological Characteristics

The clinical and pathological characteristics of 506 patients are presented in **Table 1**. According to the definition of MetS, there were 153 patients with MetS (30.20%) and 388 without MetS (69.80%). Out of the total number of patients, 39.70% had hyperglycemia and 56.30% had a BMI ≥25 kg/m^2^. In addition, the percentage of patients with hypertension and dyslipidemia was 41.30% and 49.60%, respectively. According to the different MetS components, EC patients were characterized as follows: 0 components for 73 cases (14.4%), 1–2 components for 280 cases (55.3%) and ≥3 components for 153 cases (30.2%). Lymph node metastasis (LNM) positive, Lymph-vascular space invasion (LVSI) positive, deep-myometrial infiltration (MI), were found in 11.1, 17.2, and 33.4% of patients, respectively (**Table 1**).

**Table 1:** Clinical and pathological characteristics for 506 EC patients..

Characteristics	Number of patients(%)
Age	55.76 ± 9.56
<55 years	220 (43.48)
≥55 years	286 (56.52)
Menopause	
Premenopausal status	184 (36.40)
Postmenopausal status	322 (63.60)
Histotype	
EEA	436 (86.20)
SEA	70 (13.80)
Grade	
1	169 (33.40)
2–3	337 (66.60)
Stage	
I	402(79.40)
II–IV	104 (20.60)
LNM	
Negative	364 (71.9)
Positive	56 (11.1)
NA	86 (17)
LVSI	
Negative	419 (82.8)
Positive	87 (17.2)
MI	
Superficial	337 (66.6)
Deep	169 (33.4)
Ascites tumor	
Negative	352 (69.6)
Positive	35 (6.9)
NA	119 (23.5)
MetS	
Without	353 (69.80)
With	153 (30.20)
MetS components	
0 components	73 (14.4)
1–2 components	280 (55.3)
≥3 components	153 (30.2)
Blood glucose	
Normal glycemia	305 (60.30)
Hyperglycemia	201 (39.70)
BMI	
<25 kg/m^2^	221 (43.70)
≥25 kg/m^2^	285 (56.30)
Hypertension	
Without	297 (58.70)
With	209 (41.30)
Dyslipidemia	
Without	255 (50.40)
With	251 (49.60)
TG	
<1.69 mmol/l	339 (67%)
≥1.69 mmol/l	167 (33%)
HDL-C	
≥1.0 mmol/l	369 (72.92%)
<1.0 mmol/l	137 (27.08%)

### Association Between MetS and Clinicopathological Characteristics

Further, we analyzed the association between MetS and clinicopathological characteristics (**Table 2**). The results showed that patients with MetS had more elderly (≥55 years, 71.24% vs 50.14%, *P <*0.05) and postmenopausal proportion (72.55% vs 59.77%, *P <*0.05) than patients without MetS. The proportion of MetS patients with high grade (2–3) and advanced stage (II–IV) EC was greater than patients without MetS (30.72% vs 20.40%, 33.99% vs 14.73%, *P <*0.05). In addition, patients with MetS had higher positive rate of LNM, LVSI, and deep-MI proportion (25.98% vs 7.85%, 24.84% vs 13.88%, 44.44% vs 28.61%, *P <*0.05). These results suggested that EC patients with MetS have increased tumor aggressiveness.

**Table 2:** MetS associated with clinicopathological characteristics of EC patients.

Characteristics	Without MetS	With MetS	P-value
	353 (69.80%)	153 (30.20%)	
Age			**0.000**
<55 years	176 (49.86)	44 (28.76)	
≥55 years	177 (50.14)	109 (71.24)	
Menopause			**0.006**
Premenopausal status	142 (40.23)	42 (27.45)	
Postmenopausal status	211 (59.77)	111 (72.55)	
Histotype			0.175
EEA	309 (87.54)	127 (83.01)	
SEA	44 (12.46)	26 (16.99)	
Grade			**0.012**
1	281 (79.60)	106 (69.28)	
2–3	72 (20.40)	47 (30.72)	
Stage			**0.000**
I	301 (85.27)	101 (66.01)	
II–IV	52 (14.73)	52 (33.99)	
LNM			**0.000**
Negative	270 (92.15)	94 (74.02)	
Positive	23 (7.85)	33 (25.98)	
LVSI			**0.003**
Negative	304 (86.12)	115 (75.16)	
Positive	49 (13.88)	38 (24.84)	
MI			**0.001**
Superficial	252 (71.39)	85 (55.56)	
Deep	101 (28.61)	68 (44.44)	
Ascites tumor			0.904
Negative	248 (90.84)	104 (91.23)	
Positive	25 (9.16)	10 (8.77)	

The bold values means the P-value < 0.05.

### Effects of MetS and its Components on OS and RFS of EC Patients

**Figure 1:**
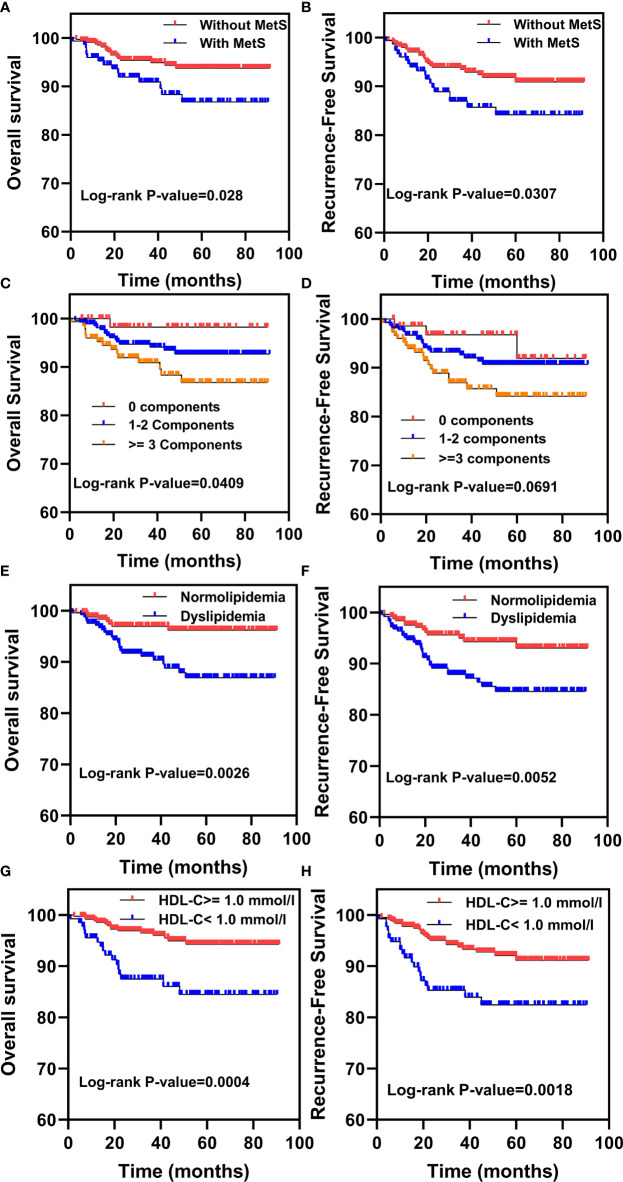
Kaplan–Meier analysis for overall survival (OS) and recurrence-free survival (RFS) in EC patients with MetS and its components. **(A, B)** The Kaplan–Meier survival analysis of the OS and RFS between EC patients with or without MetS. **(C, D)** Kaplan–Meier survival analysis of the OS and RFS between EC patients with 0 components, 1–2 components or ≥3 components. **(E, F)** The Kaplan–Meier survival analysis of the OS and RFS between EC patients with normolipidemia and dyslipidemia. **(G, H)** The Kaplan–Meier survival analysis of the OS and RFS between EC patients with HDL-C ≥1.0mmol/l and HDL-C <1.0mmol/l.

To explore the effects of MetS and its components on OS and RFS in EC patients, we firstly performed the univariate analysis as shown in **Table 3**. Results indicated that MetS was closely related to OS (HR = 2.14; 95%CI: 1.07–4.28; P = 0.032) and RFS (HR = 1.80; 95%CI: 1.0–3.3; P = 0.045) of EC patients. The K–M analysis showed that EC patients with MetS had shorter OS and RFS rates compared to patients without MetS (**Figures 1A, B**). The OS time decreased in patients who had ≥3 components vs 1–2 or 0 components (P = 0.045), while there was no apparent difference observed for RFS rates (P = 0.0691) (**Figures 1C, D**). In addition, there was a significant correlation between OS and dyslipidemia (HR = 3.20; 95%CI: 1.44–7.12; P = 0.004), HDL-C <1.0 mmol/l (HR = 3.24; 95%CI: 1.62–6.49; P = 0.0009). EC patients with dyslipidemia or HDL-C <1.0 mmol/l had shorter OS and RFS rates compared to EC patients with normolipidemia or an HDL-C ≥1.0 mmol/l (**Figures 1E–H**). Patients with dyslipidemia and an HDL-C < 1.0 mmol/l also were more likely to have recurrence. Using univariate analysis, we also found that age, histotype, tumor grade, and tumor stage were associated with OS and RFS. Altogether, these results suggested that MetS was associated with poor prognosis in EC patients. Among the MetS components, dyslipidemia, especially an HDL-C <1.0mmol/l, was significantly correlated with poor prognosis in EC patients.

**Table 3:** Univariate analysis of OS and RFS for EC patients.

Variable	OS			RFS		
	HR	95%CI	P-value	HR	95%CI	P-value
Age						
<55 years	1.0	Ref		1.0	Ref	
≥55 years	2.87	1.24–6.65	**0.014**	2.1	1.1–4.1	**0.025**
Menopause						
Premenopausal status	1.0	Ref		1.0	Ref	
Postmenopausal status	1.52	0.70–3.28	0.291	2.0	1.0–4.0	0.055
Histotype						
Type I	1.0	Ref		1.0	Ref	
Type II	9.02	4.50–18.09	**<0.001**	8.8	4.9–16.0	**<0.001**
Grade						
1	1.0	Ref		1.0	Ref	
2–3	13.92	6.02–32.20	**<0.001**	11.9	6.0–23.7	**<0.001**
Stage						
I	1.0	Ref		1.0	Ref	
II–IV	16.41	7.09–37.96	**0.001**	10.0	5.3–19.0	**<0.001**
MetS						
No	1.0	Ref		1.0	Ref	
Yes	2.14	1.07–4.28	**0.032**	1.8	1.0–3.3	**0.045**
MetS components						
0 components	1.0	Ref		1.0	Ref	
1–2 components	4.06	0.54–30.63	0.174	1.9	0.6–6.3	0.305
≥3 components	7.36	0.97–55.70	0.053	3.1	0.9–10.6	0.066
Blood glucose						
Normal glycemia	1.0	Ref		1.0	Ref	
Hyperglycemia	1.06	0.8747	0.875	1.1	0.6–2.0	0.824
BMI						
<25 kg/m^2^	1.0	Ref		1.0	Ref	
≥25 kg/m^2^	1.31	0.64–2.68	0.458	1.4	0.7–2.5	0.308
Hypertension						
Without	1.0	Ref		1.0	Ref	
With	1.30	0.65–2.61	0.457	1.1	0.6–2.0	0.728
Dyslipidemia						
Without	1.0	Ref		1.0	Ref	
With	3.20	1.44–7.12	**0.004**	2.6	1.3–4.9	**0.004**
TG						
<1.69 mmol/l	1.0	Ref		1.0	Ref	
≥1.69 mmol/l	0.92	0.43–1.94	0.820	0.8	0.4–1.6	0.617
HDL-C						
≥1.0 mmol/l	1.0	Ref		1.0	Ref	
<1.0 mmol/l	3.24	1.62–6.49	**0.0009**	2.6	1.5–4.8	**0.001**

The bold values means the P-value < 0.05.

Cox multivariate analysis showed associations between MetS and its components and OS or RFS after adjusting for basic factors (**Tables 4**, **5**). These results indicated that dyslipidemia and HDL-C <1.0mmol/l were significantly associated with worse OS and RFS after adjusting for age. However, after adjusting for age, histotype, grade, and stage, only HDL-C was associated with an increased risk of EC-related deaths (HR = 2.2, 95%CI: 1.1–4.4; P = 0.034), and there was no significant difference observed for RFS. These results suggested that among MetS components, HDL-C was an independent risk factor for EC.

**Table 4:** Cox multivariate analysis of OS for MetS and its components in EC patients.

Variable	OS-adjust I			OS-adjust II		
	HR	95%CI	P-value	HR	95%CI	P-value
MetS						
Without	1.0	Ref		1.0	Ref	
With	1.80	0.9–3.7	0.097	1.3	0.6–2.6	0.518
BMI						
<25 kg/m^2^	1.0	Ref		1.0	Ref	
≥25 kg/m^2^	1.3	0.6–2.6	0.497	1.5	0.7–3.0	0.301
Hypertension						
Without	1.0	Ref		1.0	Ref	
With	1.1	0.5–2.1	0.890	1.2	0.6–2.5	0.574
Blood glucose						
Normal glycemia	1.0	Ref		1.0	Ref	
Hyperglycemia	0.9	0.4–1.8	0.675	1.0	0.5–2.1	0.960
Dyslipidemia						
Without	1.0	Ref		1.0	Ref	
With	3.1	1.4–6.9	**0.006**	1.6	0.7–3.7	0.246
TG						
<1.69 mmol/l	1.0	Ref		1.0	Ref	
≥1.69 mmol/l	0.9	0.4–1.8	0.714	0.76	0.6	0.3–1.2
HDL-C						
≥1.0 mmol/l	1.0	Ref		1.0	Ref	
<1.0 mmol/l	3.6	1.8–7.2	**<0.001**	2.2	1.1–4.4	**0.034**
MetS components						
0 components	1.0	Ref		1.0	Ref	
1–2 components	3.4	0.4–25.6	0.241	5.9	0.8–45.4	0.088
≥3 components	5.4	0.7–41.7	0.105	6.0	0.8–46.7	0.088

Adjust I for: Age.

Adjust II for: Age, Histotype, Grade, Stage.

The bold values means the P-value < 0.05.

**Table 5:** Cox multivariate analysis of RFS for MetS and its components in EC patients.

Variable	RFS-adjust I			RFS-adjust II		
	HR	95%CI	P-value	HR	95%CI	P-value
MetS						
Without	1.0	Ref		1.0	Ref	
With	1.6	0.9–3.0	0.120	1.09	0.58–2.03	0.792
BMI						
<25 kg/m^2^	1.0	Ref		1.0	Ref	
≥25 kg/m^2^	1.4	0.7–2.5	0.333	1.61	0.86–3.00	0.133
Hypertension						
Without	1.0	Ref		1.0	Ref	
With	0.9	0.5–1.7	0.830	1.02	0.55–1.89	0.956
Blood glucose						
Normal glycemia	1.0	Ref		1.0	Ref	
Hyperglycemia	0.9	0.5–1.7	0.755	1.00	0.54–1.85	0.995
Dyslipidemia						
Without	1.0	Ref		1.0	Ref	
With	2.5	1.3–4.8	**0.006**	1.38	0.71–2.74	0.337
TG						
<1.69 mmol/l	1.0	Ref		1.0	Ref	
≥1.69 mmol/l	0.8	0.4–1.6	0.535	0.58	0.30–1.13	0.110
HDL-C						
≥1.0 mmol/l	1.0	Ref		1.0	Ref	
<1.0 mmol/l	2.8	1.5–5.0	**<0.001**	1.65	0.90–3.02	0.105
MetS components						
0 components	1.0	Ref		1.0	Ref	
1–2 components	1.6	0.5–5.5	0.429	2.90	0.84–9.97	0.091
≥3 components	2.5	0.7–8.6	0.153	2.61	0.74–9.18	0.134

Adjust I for: Age.

Adjust II for: Age, Histotype, Grade, Stage.

The bold values means the P-value < 0.05.

### ROC Analysis and Construction of a Nomogram

**Figure 2:**
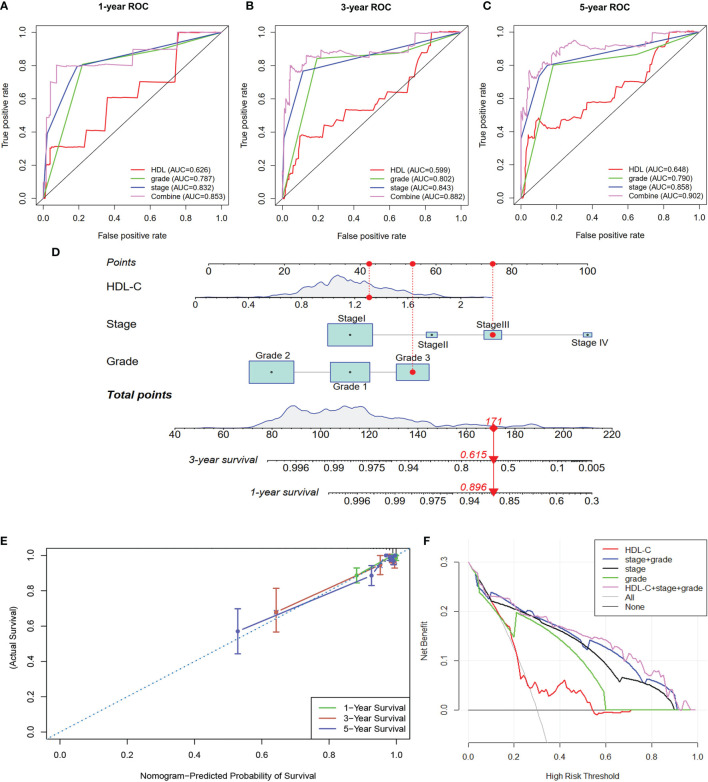
(**A–C**) ROC analysis were performed to evaluate the ability of HDL-C in predicting EC patient prognosis at 1-, 3-, and 5-years. (**D**) A nomogram was constructed based on HDL-C, grade, and stage to predict 1- and 3-year survival rates of EC patients. (**E, F**) Calibration curve analysis and DCA analysis of the nomogram.

To further evaluate the ability of HDL-C in predicting EC patient prognosis, we performed ROC analysis as shown in **Figures 2A–C**. These results showed that the area under curve (AUC) of HDL-C was 0.626, 0.599, and 0.648 at 1-, 3-, and 5-years, respectively. It is important to note that the AUC of combine factors (HDL-C + grade + stage) was 0.853, 0.882, and 0.902 at 1-, 3-, and 5-years, respectively, which was better than any single factor. This suggests that this combination better predicts the prognosis of EC patients compared to using traditional stage or grade.

Nomograms are used for multiple-parameter diagnosis or to predict tumorigenesis or development (15). To provide clinicians with a method to quantitatively predict the prognosis of EC patients, we constructed a nomogram based on HDL-C, grade, and stage to predict 1- and 3-year survival rates of EC patients (**Figure 2D**). A total number of points was calculated for each patient based on these different parameters. The higher the total number, the worse the prognosis of the patient. Furthermore, calibration curve analysis showed that the nomogram-predicted probability of survival was closed to actual survival at 1-, 3-, and 5 years (**Figure 2E**). The DCA analysis also indicated that the combine factors (HDL-C + grade + stage) showed a better ability to predict survival compared to HDL-C, stage, grade or stage + grade (**Figure 2F**). Taken together, this constructed nomogram can better predict EC patient survival.

## Discussion

In this retrospective study, we explored the association between MetS and clinicopathological characteristics of EC patients. Also, the effect of MetS and its components on the prognosis of EC was studied. We found that MetS was positively related to several clinicopathological characteristics, such as tumor grade, stage, LNM, etc. Our results also indicated that MetS was associated with a 2.14-fold increased risk of death and a 1.8-fold increased risk of recurrence in EC patients, although these correlations were not significant using the multivariate-adjust model. Among the MetS components, only HDL-C was found to be associated with OS of EC patients in the multivariate-adjust model. Then, a nomogram combining HDL-C, grade, and stage was constructed to predict prognosis. To our knowledge, this retrospective study is the first to explore the association between MetS and its components on the prognosis of EC patients in a Chinese population, and the nomogram constructed has good ability to predict survival of EC patients.

It is known that MetS, hyperglycemia, obesity, hypertension, and dyslipidemia are high-risk factors for cardiovascular and other diseases. In addition, a great number of studies confirmed that MetS is associated with cancer development and cancer-related mortality (16). A meta-analysis, including 38,940 cases, showed that although ages, populations, and the definitions of MetS differed, MetS was associated with increased cancer risk. These cancers included liver, colorectal and bladder cancers in men and breast and colorectal cancers in women (17). A 14-year follow-up study showed that MetS was associated with a 56% greater age-adjusted risk in cancer mortality (18). However, the role of MetS in some cancers remains controversial. One study reported that there was no correlation between MetS and renal cell carcinoma. In contrast, another study reported that there was a slight correlation between MetS and renal cell carcinoma, which may be a result of the population size used in this study versus the other study showing no correlations (19, 20). Also, MetS was found to be associated with increased risk for EC. Our study found that MetS was closely associated with advanced stage, high grade, positive LNM, positive LVSI, and deep MI, suggesting that MetS may contribute to increased aggressiveness of tumors. It was reported that the prevalence of MetS in postmenopausal women with endometrial hyperplasia and EC was higher than what was observed in premenopausal women (21). Furthermore, our study found that patients with MetS had more elder and postmenopausal proportion compared to patients without MetS, which is consistent with previous studies.

Even though many studies explored the relationship between MetS and the risk of various cancers, there are few studies investigating the relationship between MetS and cancer mortality. Esposito et al. showed that MetS was associated with an increased risk and mortality of colon cancer in both men and women (11). A SEER database study also reported that MetS was associated with a lower cancer-specific survival in early stage EC cases (HR = 1.28, 95%CI: 1.09–1.53) (16). Besides, patients with 1 or 2 MetS components have worse survival rates compared to those with 0 components, based on a breast cancer study (22). However, there are no reports understanding the relationship between MetS components and OS in EC. In our study, univariate analysis showed that compared with people without MetS, EC patients with MetS showed significant correlations with both OS and RFS. Among the components of MetS, dyslipidemia and HDL-C were also related with OS and RFS in EC patients. In addition, K–M survival analysis showed that patients with MetS, dyslipidemia, and HDL-C levels <1.0 mmol/l showed lower OS and RFS rates compared to patients without MetS, dyslipidemia, and HDL-C levels ≥1.0 mmol/l, respectively. Interestingly, with the number of MetS components increased, the OS of patients with EC decreased. We found that patients with ≥3 components showed shorter OS rates compared to patients with 0 or 1–2 components. Our results are consistent with conclusions reported in previous studies, suggesting the most severe the metabolism disorder, the worse the prognosis of EC patients.

The mechanism behind MetS in promoting EC remains unclear, although it may be attributed to long-term hyperglycemia, obesity, dyslipidemia, insulin, and inflammatory cytokines (23). Previous studies showed that insulin resistance/hyperinsulinemia, abnormal endogenous estrogen signaling, inflammatory cytokines, and adipocytokines (IL-6, TNF-α, adiponectin, visfatin, and leptin) may be the main mechanisms behind obesity that is associated with EC (24). Epidemiological studies indicate that obesity-related insulin resistance is a potential risk factor for EC. Insulin and insulin-like growth factor-1 promote the proliferation and migration of EC cells through the PI3K/Akt and RAS/MAPK pathways (9). Decreased serum adiponectin levels or increased visfatin levels are independent risk factors for EC. The ratio of visfatin to adiponectin has a certain reference value for the diagnosis of EC. Adiponectin may activate the expression of the downstream LKB1-AMPK/S6 signal axis by binding to AdipoRs, thereby inhibiting the proliferation, adhesion and invasion of EC cells (25). Epidemiological studies also indicated that there was a significant correlation between diabetes mellitus and the incidence of EC. It is important to note that this association remained after adjusting for obesity (26). The exact molecular mechanisms behind the association between diabetes and cancer is not clear. However, some studies confirmed that hyperglycemia, the insulin/insulin-like growth factor (IGF) axis and inflammatory cytokines play important roles in promoting EC proliferation and invasion (27). Hyperglycemia may also directly promote hyperinsulinemia and indirectly induce tumor development by increasing IGF-1 function. In addition, an increasing number of studies found that metformin, an antidiabetic drug, inhibited the growth of EC by inhibiting the AMPK and PI3K/Akt/mTOR signaling pathways (28). Even though previous work showed that each component of MetS is associated with cancer development, it is not known whether these effects are additive or synergistic. It has been reported that visfatin upregulated the expression of insulin receptor (IR) and insulin receptor substrate (IRS) 1/2, both of which cooperated with insulin to activate the PI3K/Akt and MAPK/ERK1/2 signaling pathways, thereby promoting proliferation of endometrial cancer cell and inhibiting apoptosis in EC. In addition, obesity, hyperlipidemia, and hyperglycemia, as important pathogenic factors of MetS, promoted the occurrence and development of malignant tumors by inducing insulin resistance (29). Altogether, multiple molecular mechanisms associated with MetS may be closely related to the increased risk and deaths associated with EC.

To explore the key factors associated with EC in relation to MetS components, multivariate analysis was performed. In addition to MetS and its components, there was an association between age, histotype, grade, and stage associated with OS and RFS in EC patients. Therefore, age, histotype, grade, and stage were adjusted. In the multivariate-adjust model, we found that only HDL-C was associated with an increased risk for death related to EC. Meanwhile, MetS did not show a significant correlation EC death even though it was reported that MetS is an independent prognostic factor for EC patients in a study including 385 cases (18). Differences in conclusions may be explained by sample sizes used in these studies. One study reported that the TG/HDL-c ratio may be a potential marker for EC (30). Furthermore, ROC analysis found that a combination of factors (HDL-C + grade + stage) better predict EC patient prognosis in comparing to stage or grade. It is known that tumor grade and stage are important factors to evaluate the prognosis of EC patients (31). However, other important factors, such as metabolic disorders, are also closely related to patient survival and prognosis. More and more nomogram prognostic models have been established to predict the prognosis of patients (32). In our study, we used R software to generate a nomogram based on stage, grade, and HDL-C, so as to intuitively and visually predicting the survival of patients with EC. The calibration curves also showed that in the nomogram the predicted value had high consistency with the actual value. DCA is a simple method used to assess the feasibility and benefit of prediction tools (33). In our study, the DCA confirmed that our nomogram model was superior to stage, grade, HDL-C and stage + grade when it comes to predicting the survival of EC patients.

However, our study faced several limitations. First, waist circumference information was not recorded in our study, which limits the analysis of different definitions of MetS and EC. We defined MetS according to the Chinese Diabetes Society standard from 2004, which is suitable for Chinese population characteristics. Second, further studies with a larger sample size are needed to confirm our results. Similarly, the nomogram we constructed needed to be validated in other cohorts. Lastly, the molecular mechanisms behind these relationships need. to be further explored.

In conclusion, through a retrospective study of 506 endometrial cancers, our study indicated that MetS is closely related to clinicopathological characteristics. In addition, MetS and its components such as dyslipidemia, was associated with poor outcomes in EC patients. The prevalence of the individual components of MetS increases with worse outcomes in EC patients. Furthermore, a nomogram combined HDL-C, grade, and stage was constructed and the nomogram has good ability to predict EC patient survival. Our study supports that improving MetS is expected to improve the prognosis of patients with EC.

## Data Availability Statement

The raw data supporting the conclusions of this article are available from the corresponding author upon request

## Ethics Statement

The studies involving human participants were reviewed and approved by the Peking University People’s Hospital. The patients/participants provided their written informed consent to participate in this study. Written informed consent was obtained from the individual(s) for the publication of any potentially identifiable images or data included in this article.

## Author Contributions

XY: Project development, analysis and manuscript writing. XL: Project development and data analysis. YD, YF, YC, LZ, and SZ: Data collection. JW and JZ: Project development, supervision and manuscript revising. All authors contributed to the article and approved the submitted version.

## Funding

This work was supported by the National Key Technology R&D Program of China (Nos. 2019YFC1005200 and 2019YFC1005201), the Natural Science Foundation of Beijing (No. 7202213) and the National Natural Science Foundation of China (No. 82072861, 81672571, and 81874108).

## Conflict of Interest

The authors declare that the research was conducted in the absence of any commercial or financial relationships that could be construed as a potential conflict of interest.

## Publisher’s Note

All claims expressed in this article are solely those of the authors and do not necessarily represent those of their affiliated organizations, or those of the publisher, the editors and the reviewers. Any product that may be evaluated in this article, or claim that may be made by its manufacturer, is not guaranteed or endorsed by the publisher.
